# Prevalence, antibiotic susceptibility and virulence factors of *Enterococcus* species in racing pigeons (*Columba livia f. domestica*)

**DOI:** 10.1186/s12917-019-2200-6

**Published:** 2020-01-08

**Authors:** Beata Dolka, Michał Czopowicz, Dorota Chrobak-Chmiel, Aleksandra Ledwoń, Piotr Szeleszczuk

**Affiliations:** 1 Department of Pathology and Veterinary Diagnostics, Institute of Veterinary Medicine, Warsaw University of Life Sciences – SGGW, Nowoursynowska 159c St, 02-776 Warsaw, Poland; 2 Division of Veterinary Epidemiology and Economics, Institute of Veterinary Medicine, Warsaw University of Life Sciences-SGGW, Nowoursynowska 159c St, 02-776 Warsaw, Poland; 3 Department of Preclinical Sciences, Institute of Veterinary Medicine, Warsaw University of Life Sciences-SGGW, Ciszewskiego 8 St, 02-786 Warsaw, Poland

**Keywords:** *Enterococcus*, Racing pigeons, Virulence factors, Antimicrobial resistance, VRE

## Abstract

****Background**:**

This study was aimed to investigate the intestinal microbiota in racing pigeons with regard to *Enterococcus* species distribution, virulence factors and antibiotic susceptibility. Three methods (API, Multiplex *sod*A-PCR, 16S rRNA sequencing) were compared for *Enterococcus* species identification. Cloacal samples from 179 apparently healthy pigeons of 13 different flocks were tested.

**Results:**

Multiplex *sod*A-PCR and 16S rRNA gene sequencing showed almost perfect agreement in *Enterococcus* species identification. Isolates were identified as *Enterococcus columbae *(34.5%), *Enterococcus hirae* (20.7%), *Enterococcus faecalis* (11.7%), *Enterococcus faecium* (11.7%), *Enterococcus gallinarum* (9%), *Enterococcus mundtii* (4.8%), *Enterococcus casseliflavus* (3.4%), *Enterococcus cecorum* (2.1%), *Enterococcus durans* (2.1%). More *Enterococcus* species were found after the race season than before. The study showed differences between *Enterococcus* species in relation to 68.8% (22/32) biochemical parameters. Six out of seven virulence genes were detected: *gelE* (43.5%)*, asa1* (42.1%), *efaA* (30.3%), *ace* (30.3%), *cylA* (27.6%), and *esp* (9%). None of the isolates harboured *hyl* gene. Overall 15.2% of *Enterococcus* isolates produced gelatinase, but 66.7% *gelE* genes were silent. *Enterococcus faecalis* showed the most often *efaA*, *ace* and gelatinase activity than other enterococcal species. Nearly all isolates (93.1%) were resistant to at least one antibiotic. The most frequent resistance was to enrofloxacin (80%), doxycycline with teicoplanin (73.1%), erythromycin (49.7%). The study revealed significant differences between some enterococcal species in the antibiotic susceptibility to different antibiotics. *Enterococcus columbae* and *E. cecorum* showed significantly more frequent resistance to chloramphenicol than other enterococci. The presence of VRE (19.3%), HLGR (2.8%) and no LRE were found. Overall 30.3% of isolates were positive for vancomycin resistance genes, where *van*C1 (*E. gallinarum*), *van*C2-C3 (*E. hirae*, *E. casseliflavus*), *van*B (*E. columbae*) predominated.

****Conclusions**:**

We conclude, that intestinal microbiota in racing pigeons is composed by 9 different *Enterococcus* species. Given that racing pigeons are kept in close contact with humans and backyard animals, combined with their long-distance flight abilities, they can serve as potential source of virulent and antibiotic resistant *Enterococcus* spp. in the environment.

## Background

Among known 58 *Enterococcus* species, only some of them were found in pigeons [1–4]. In 1990, *Enterococcus columbae* was described for the first time in pigeons [5]. Other enterococci such as *Enterococcus cecorum*, *Enterococcus faecalis*, *Enterococcus faecium*, *Enterococcus gallinarum*, *Enterococcus casseliflavus* were recognized as the minor coccal members of the pigeon intestinal microbiota [1]. Although enterococci are usually thought to be harmless commensals, they can also act as opportunistic pathogens. In recent years, their increased role has been noted in poultry and pigeon pathology [6–9]. According to the literature the drug-resistance of the bacteria isolated from pigeons may be very high [4, 10]. Multidrug-resistant enterococci have emerged as a global threat to public health, and vancomycin-resistant enterococci (VRE) currently rank among the leading bacterial causes of nosocomial infections. Although several studies reported the antibiotic resistance in avian *Enterococcus* [8, 11, 12] and the enterococcal virulence factors in humans, poultry, farm animals and wildlife, food, environment [11, 13–17], so far little is known about the antimicrobial susceptibility of enterococci from pigeons [4, 18]. Moreover the occurrence of virulence factors has not been described among *Enterococcus* spp. in domestic pigeons. This lack of sufficient data highlights the need for investigation of *Enterococcus* from racing pigeons.

The aim of this study was to evaluate the enterococcal microbiota of racing pigeons (*Columba livia f. domestica*) by investigating the *Enterococcus* species composition in cloacal samples, the prevalence of virulence factors, antimicrobial susceptibility including resistance to vancomycin, high-level gentamicin resistance (HLGR), and linezolid-resistant enterococci (LRE).

## Results

### Prevalence of *Enterococcus* spp. in racing pigeons

A total of 145 *Enterococcus* isolates were recovered from 179 pigeons, and 138/179 pigeons were positive for enterococci. Among 131 pigeons only one isolate was retrieved from each bird, and two different species in different combinations (*E. columbae & Enterococcus mundtii* or *E. columbae & Enterococcus gallinarum*; *Enterococcus hirae & E. gallinarum* or *E. hirae & E. mundtii*) were found in 7 pigeons. The prevalence of *Enterococcus* spp. ranged from 36.8 to 100% (median value 92.9%), while 77.1% (CI 95%: 70.4, 82.6%) of the pigeons where positive for enterococci. *Enterococcus columbae* and *E. hirae* predominated in pigeons from lofts located in central Poland, while *E. faecium* predominated in pigeons in southern Poland (Additional file 1).

### Identification of *Enterococcus* species

The final results of *Enterococcus* species identification are showed in Table 1. *Enterococcus columbae* occurred significantly more often (*p* = 0.026) than other enterococci. *Enterococcus hirae* was significantly more often isolated than *E. gallinarum* and other enterococci (*p* = 0.015). *Enterococcus faecalis* and *E. faecium* were significantly more often occurred in pigeons than *E. casseliflavus* and other enterococci (*p* = 0.023). There were highly significant differences (*p* < 0.001) in the occurrence of enterococcal species between lofts. In 38.5% (5/13) lofts *E. columbae* predominated, but in 23.1% (3/13) of lofts *E. columbae* was not detected (Additional file 1).

**Table 1:** Prevalence of particular *Enterococcus* species (*n* = 145) in racing pigeons

*Enterococcus* species	Number of isolates	Prevalence (CI 95%)
*E. columbae*	50	34.5 (27.2, 42.5)
*E. hirae*	30	20.7 (14.9, 28.0)
*E. faecalis*	17	11.7 (7.5, 18.0)
*E. faecium*	17	11.7 (7.5, 18.0)
*E. gallinarum*	13	9 (5.3, 14.7)
*E. mundtii*	7	4.8 (2.4, 9.6)
*E. casseliflavus*	5	3.4 (1.5, 7.8)
*E. cecorum*	3	2.1 (0.7, 5.9)
*E. durans*	3	2.1 (0.7, 5.9)

### Biochemical characteristics

API test allowed to identification 46.2% isolates (67/145) with perfect profile (13.8%, 20/145 isolates), very good (22.1%, 32/145), and good profiles (10.3%, 15/145). The remaining isolates gave doubtful profile (9%, 13/145); perfect, very good or good identification to the genus level (10.3%, 15/145), acceptable (2.8%, 4/145), and unacceptable profiles (29.7%, 43/145) or lack of profile (2%, 3/145). Biochemical patterns of pigeon *Enterococcus* spp. were presented in Table 2. Pigeon enterococci were often positive (85–100%) for β-glucosidase (βGLU), ribose (RIB), lactose (LAC), maltose (MAL), saccharose (SAC), and rare positive (0–6%) for β-glucuronidase (βGUR), glycogen (GLYG), urease (URE).

**Table 2:** Percent of positive reactions (%) in rapid ID 32 STREP (bioMérieux, France) for *Enterococcus* species (n = 145) isolated from racing pigeons

Parameter	*E. columbae n* = 50	*E. hirae n* = 30	*E. faecalis n* = 17	*E. faecium* *n* = 17	*E. gallinarum n* = 13	*E. mundtii n* = 7	*E. casseliflavus* *n* = 5	*E. cecorum* *n* = 3	*E. durans* *n* = 3	Chi-square test *p*-value
ADH	22.0↓	93.3	100	94.1	92.3	100	40.0↓	33.3↓	100	< 0.001
βGLU	92.0	100	100	100	100	100	100	100	100	0.365
βGAR	78.0	43.3↓	5.9↓	23.5↓	38.5↓	42.9↓	20.0↓	66.7	66.7	< 0.001
βGUR	6.0	0	0	0	0	0	0	0	0	0.590
αGAL	96.0	90.0	29.4↓	70.6	92.3	100	80.0	100	66.7	< 0.001
PAL	46.0↑	3.3	0	0	7.7	0	0	66.7↑	0	< 0.001
RIB	100	100	100	100	100	85.7	100	100	100	0.624
MAN	76.0	13.3↓	100	100	100	71.4	100	100	33.3↓	< 0.001
SOR	88.0	23.3↓	88.2	29.4↓	0↓	28.6↓	60.0	66.7	33.3	< 0.001
LAC	98.0	96.7	100	100	100	100	100	100	100	0.960
TRE	96.0	80.0	100	88.2	100	57.1↓	80.0	100	100	0.019
RAF	100.0	43.3	29.4↓	47.1↓	92.3	28.6↓	80.0	100	66.7	< 0.001
VP	86.0	83.3	76.5	88.2	69.2	85.7	80.0	66.7	100	0.829
APPA	14.0	10.0	5.9	5.9	0	14.3	0	0	33.3	0.507
βGAL	90.0	93.3	17.7↓	94.1	100	100	100	66.7	100	< 0.001
PYRA	10.0↓	93.3	100	100	100	100	100	0↓	100	< 0.001
βNAG	12.0↓	83.3	88.2	70.6	100	57.1	60.0	100	33.3	< 0.001
GTA	96.0	90.0	100	52.9↓	100	85.7	80.0	100	66.7	< 0.001
HIP	2.0↓	3.3↓	41.2	41.2	92.3↑	0↓	0↓	33.3	0↓	< 0.001
GLYG	2.0	0	0	5.9	0	0	0	0	0	0.883
PUL	20.0↑	0	0	0	0	0	20.0↑	0	0	0.004
MAL	100	96.7	100	100	100	100	100	100	100	0.923
MEL	100	86.7	29.4↓	64.7↓	92.3	28.6↓	80.0	100	66.7	< 0.001
MLZ	26.0	0	82.4↑	11.8	0	0	80.0↑	33.3	0	< 0.001
SAC	100	100	88.2	100	100	100	100	100	100	0.360
LARA	76.0	16.7↓	17.7↓	94.1	100	85.7	100	33.3↓	0↓	< 0.001
DARL	48.0↑	3.3	0	0	0	0	0	66.7↑	0	< 0.001
MβDG	66.0↓	96.7	94.1	100	100	100	100	100	100	< 0.001
TAG	36.0	10.0	100↑	47.1	100↑	57.1	80.0↑	66.7↑	0↓	< 0.001
βMAN	24.0↓	63.3	94.1	70.6	100	85.7	100	100	66.7	< 0.001
CDEX	92.0	83.3	100	82.4	92.3	100	40.0↓	100	66.7	0.036
URE	2.0	0	0	5.9	0	0	0	0	0	0.883

*ADH* (arginine dihydrolase), *βGLU* (β-glucosidase), *βGAR* (β-galactosidase), *βGUR* (β-glucuronidase), *αGAL* (α-galactosidase), *PAL* (alkaline phosphatase), *RIB* (ribose), *MAN* (mannitol), *SOR* (sorbitol), *LAC* (lactose), *TRE* (trehalose), *RAF* (rafinose), *VP* (Voges Proskauer, aceton production), *APPA* (alanyl-phenylalanyl-proline arylamidase), *βGAL* (β-galactosidase), *PYRA* (pyroglutamic acid arylamidase), *βNAG* (N-acetyl-β-glucosaminidase), *GTA* (glycyl-tryptophan arylamidase), *HIP* (hydrolysis of hipurate), *GLYG* (glycogen), *PUL* (pullulane), *MAL* (maltose), *MEL* (melibiose), *MLZ* (melezitose), *SAC* (saccharose), *LARA* (L-arabinose), *DARL* (D-arabitol), *CDEX* (cyclodextrin), *MβDG* (methyl-βD-glucopyranoside), *TAG* (tagatose), *βMAN* (β-mannosidase), *URE* (urease)

### Comparison of multiplex *sodA* PCR to biochemical testing and 16S rRNA sequencing in identification of *Enterococcus* species

Table 3 shows the comparison results of Multiplex PCR and sequencing used for *Enterococcus* species identification. A total of 96.6% (140/145) isolates were identified similarly by Multiplex PCR and sequencing. There was almost perfect agreement (Cohen’s kappa = 0.956; CI 95%: 0.919, 0.993) between these techniques. The only disagreement concerned 5 isolates of 4 species (*E. hirae, E. faecium, E. mundtii, Enterococcus durans*)*,* which were sometimes recognized as *E. columbae* in sequencing. Final confirmation was performed by species-specific PCR and then *sod*A gene fragment sequencing. Phylogenetic analyses based on 16S rRNA gene and *sodA* gene sequences are shown in Additional file 2, Additional file 3.

**Table 3:** Comparison of Multiplex *sodA*-PCR and 16S rRNA sequencing in identification of *Enterococcus* species in racing pigeons

	*Enterococcus spp.*	16S Sequencing									Total *(n*)
		*E. columbae*	*E. hirae*	*E. faecium*	*E. faecalis*	*E. gallinarum*	*E. mundtii*	*E. casseliflavus*	*E. cecorum*	*E. durans*	
Multiplex PCR (*sodA*)	*E. columbae*	50	0	0	0	0	0	0	0	0	50
	*E. hirae*	2	28	0	0	0	0	0	0	0	30
	*E. faecium*	1	0	16	0	0	0	0	0	0	17
	*E. faecalis*	0	0	0	17	0	0	0	0	0	17
	*E. gallinarum*	0	0	0	0	13	0	0	0	0	13
	*E. mundtii*	1	0	0	0	0	6	0	0	0	7
	*E. casseliflavus*	0	0	0	0	0	0	5	0	0	5
	*E. cecorum*	0	0	0	0	0	0	0	3	0	3
	*E. durans*	1	0	0	0	0	0	0	0	2	3
Total (*n*)		55	28	16	17	13	6	5	3	2	145

Cohen’s kappa = 0.956 (CI 95%: 0.919, 0.993) – almost perfect agreement

Green indicates agreement in species identification between sequencing and PCR; yellow indicates a lack of agreement

The results of comparison of API and Multiplex PCR are showed in Additional file 4. API cannot identify *E. columbae* and *E. mundtii*, therefore, these two species were excluded from the comparative analysis of API with Multiplex PCR and sequencing. A total of 64.7% (57/88) isolates were identified in the same way by API and Multiplex PCR. Comparison of API and sequencing is showed in Additional file 5. Both tests identified identically 66.7% (56/84) isolates. Overall 38.6% (56/145) of isolates were correctly identified using 3 methods (API, PCR, sequencing). After exclusion *E. columbae* and *E. mundtii*, 63.7% (56/88) isolates were recognized correctly by 3 methods. The validity of 3 methods is presented in Additional file 6. Based on the analyzed data, the sensitivity seems to be more important parameter*.*

### Agreement analysis between GenBank and EzTaxon in identification of *Enterococcus* species

GenBank and EzTaxon identified identically all isolates to adequate *Enterococcus* species (based on 16S rRNA gene fragment). However, comparison of the percentage of identity results between these two services showed fair agreement: cut-off = 98.65%, Gwet’s AC1 = 35.0% (CI 95%: 21.7, 48.4%). GenBank gave as many as 90.3% (131/145) positive, EzTaxon only 53.1% positive (77/145).

### Detection of virulence factors

*Enterococcus* isolates of pigeon origin harboured 6 of 7 (85.7%) tested virulence genes: *gelE* (43.5%)*, asa1* (42.1%), *efaA* (30.3%), *ace* (30.3%), *cylA* (27.6%), *esp* (9%). All isolates were negative for *hyl*. In total 52.4% (69/145) of isolates had at least one virulence gene. Among positive isolates, the majority (18.6%; 27/145) harboured 4 virulence genes. Two isolates (1.4%; 2/145) contained 6 virulence genes. Differences in distribution of virulence factors among 9 enterococcal species are shown in Table 4. *EfaA* and *ace* were found significantly more often only in *E. faecalis* (*p* < 0.001). Among 9 enterococcal species *asa1* was present more often in *E. columbae* (29.5%; 18/61), *gelE* in *E. columbae* (33.3%; 21/63), *ace* in *E. columbae* (31.8%; 14/44), *efaA* in *E. faecalis* (36.4%; 16/44), *cylA* in *E. faecalis* (25%; 10/40), *esp* in *E. hirae* (30.8%; 4/13). Among isolates positive for *asa1* & *gelE* (35.2%; 51/145) majority contained also *afaA* & *ace* (53%; 27/51) or *esp & cylA* (15.7%; 8/51).

**Table 4:** Prevalence [n, (%)] of the virulence factors in different *Enterococcus* species isolated from racing pigeons

*Enterococcus* spp.	No. of isolates	Genes encoding virulence factors						
		*asa1* (aggregation substance)	*gelE* (gelatinase)	*hyl* (hyaluronidase)	*efaA* (endocarditis antigen)	*ace* (collagen-binding protein)	*esp* (enterococcal surface protein)	*cylA* (cytolysin)
*E. columbae*	50	18 (36%)	21 (42%)	0 (0%)	12 (24%)	14 (28%)	2 (4%)	9 (18%)
*E. hirae*	30	8 (26.7%)	8 (26.7%)	0 (0%)	7 (23.3%)	7 (23.3%)	4 (13.3%)	9 (30%)
*E. faecium*	17	8 (47.1%)	7 (41.2%)	0 (0%)	4 (23.5%)	4 (23.5%)	2 (11.8%)	4 (23.5%)
*E. faecalis*	17	13 (76.5%)	13 (76.5%)	0 (0%)	16 (94.1%)*↑	13 (76.5%)*↑	2 (11.8%)	10 (58.8%)
*E. gallinarum*	13	7 (53.9%)	6 (46.2%)	0 (0%)	3 (23.1%)	3 (23.1%)	2 (15.4%)	3 (23.1%)
*E. mundtii*	7	3 (42.9%)	2 (28.6%)	0 (0%)	0 (0%)	0 (0%)	0 (0%)	1 (14.3%)
*E. casseliflavus*	5	2 (40%)	3 (60%)	0 (0%)	1 (20%)	1 (20%)	1 (20%)	2 (40%)
*E. cecorum*	3	1 (33.3%)	2 (66.7%)	0 (0%)	0 (0%)	1 (33.3%)	0 (0%)	1 (33.3%)
*E. durans*	3	1 (33.3%)	1 (33.3%)	0 (0%)	1 (33.3%)	1 (33.3%)	0 (0%)	1 (33.3%)
Total	145	61 (42.1%)	63 (43.5%)	0 (0%)	44 (30.3%)	44 (30.3%)	13 (9%)	40 (27.6%)

* *p* < 0.001

Phenotypically 15.2% (22/145) of isolates showed gelatinase activity; 33.3% (21/63) of *gelE* positive isolates showed gelatinase activity, and 66.7% (42/63) of *gelE* genes were silent*.* Gelatinase production was significantly more often in *E. faecalis* (*p* < 0.001) than in other enterococci (*p* = 0.817) (Table 5). The difference in the production of gelatinase was already evident at the first day of observation.

**Table 5:** Results for gelatinase production by enterococci isolated from racing pigeons

*Enterococcus* spp.	No. of isolates	Gelatinase production				
		at 1 day	at 7 day	at 10 day	at 14 day	Overall
*E. columbae*	50	2 (4%)	4 (8%)	5 (10%)	5 (10%)	5 (10%)
*E. hirae*	30	0 (0%)	1 (3.3%)	1 (3.3%)	1 (3.3%)	1 (3.3%)
*E. faecium*	17	1 (5.9%)	2 (11.8%)	2 (11.8%)	2 (11.8%)	2 (11.8%)
*E. faecalis*	17	8 (53.3%)*↑	12 (70.6%)*↑	12 (70.6%)*↑	12 (70.6%)*↑	12 (70.6%)*↑
*E. gallinarum*	13	1 (7.7%)	1 (7.7%)	1 (7.7%)	1 (7.7%)	1 (7.7%)
*E. mundtii*	7	0 (0%)	0 (0%)	0 (0%)	0 (0%)	0 (0%)
*E. casseliflavus*	5	0 (0%)	1 (20%)	1 (20%)	1 (20%)	1 (20%)
*E. cecorum*	3	0 (0%)	0 (0%)	0 (0%)	0 (0%)	0 (0%)
*E. durans*	3	0 (0%)	0 (0%)	0 (0%)	0 (0%)	0 (0%)
Total	145	12 (8.4%)	21 (14.5%)	22 (15.2%)	22 (15.2%)	22 (15.2%)

* *p* < 0.001

### Antibiotic susceptibility of *Enterococcus* spp.

Table 6 shows the results of antibiotic susceptibility testing. The highest susceptibility was to ampicillin (100%), amoxicillin/clavulanic acid (99.3%), high-level gentamicin (97.2%), penicillin and linezolid (96.6%). The highest intermediate susceptibility was to tetracycline (62.1%), vancomycin (27.6%), enrofloxacin (20%). *Enterococcus faecium* and *E. mundtii* were rare resistant to doxycycline and teicoplanin (*p* = 0.003), *E. gallinarum* and *E. cecorum* to nitrofurantoin (*p* < 0.001), *E. casseliflavus* and *E. mundtii* to erythromycin (p < 0.001). *Enterococcus hirae, E. faecium* and *E. durans* showed significantly more frequent resistance to nitrofurantoin, and *E. faecalis* to erythromycin (p < 0.001). The resistance to chloramphenicol was significantly more often in *E. cecorum* (*p* = 0.045) and *E. columbae* (*p* = 0.040). There were no significant differences between enterococcal species in susceptibility to penicillin (*p* = 0.178), tetracycline (*p* = 0.096), high-level gentamicin (*p* = 0.575), vancomycin (*p* = 0.070), enrofloxacin (*p* = 0.443).

**Table 6:** Antibiotic susceptibility observed in commensal enterococci isolated from racing pigeons [n, (%)]

*Enterococcus* spp. (n)	Amoxicillin/ clavulanic acid			Ampicillin			Penicillin			Enrofloxacin			Doxycycline			Tetracycline			Nitrofurantoin		
	R	I	S	R	I	S	R	I	S	R	I	S	R	I	S	R	I	S	R	I	S
*E. columbae* (*n* = 50)	0 (0)	0 (0)	50 (100)	0 (0)	0 (0)	50 (100)	1 (2)	0 (0)	49 (98)	44 (88)	6 (12)	0 (0)	39 (78)	0 (0)	11 (22)	7 (14)	30 (60)	13 (26)	5 (10)	1 (2)	44 (88)
*E. hirae* (*n* = 30)	0 (0)	0 (0)	30 (100)	0 (0)	0 (0)	30 (100)	4 (13.3)	0 (0)	26 (86.7)	21 (70)	9 (30)	0 (0)	20 (66.7)	0 (0)	10 (33.3)	10 (33.3)	9 (30)	11 (36.7)	17 (56.7)*↑	6 (20)	7 (23.3)
*E. faecium* (*n* = 17)	0 (0)	0 (0)	17 (100)	0 (0)	0 (0)	17 (100)	0 (0)	0 (0)	17 (100)	13 (76.5)	4 (23.5)	0 (0)	7 (41.2)*↓	0 (0)	10 (58.8)	3 (17.6)	13 (76.5)	1 (5.9)	14 (82.4)*↑	0 (0)	3 (17.7)
*E. faecalis* (*n* = 17)	0 (0)	0 (0)	17 (100)	0 (0)	0 (0)	17 (100)	0 (0)	0 (0)	17 (100)	15 (88.2)	2 (11.8)	0 (0)	17 (100)	0 (0)	0 (0)	5 (29.4)	12 (70.6)	0 (0)	2 (11.8)	2 (11.8)	13 (76.5)
*E. gallinarum* (*n* = 13)	0 (0)	1 (7.7)	12 (92.3)	0 (0)	0 (0)	13 (100)	0 (0)	0 (0)	13 (100)	9 (69.2)	4 (30.8)	0 (0)	12 (92.3)	0 (0)	1 (7.7)	0 (0)	13 (100)	0 (0)	0 (0)*↓	0 (0)	13 (100)
*E. mundti* (*n* = 7)	0 (0)	0 (0)	7 (100)	0 (0)	0 (0)	7 (100)	0 (0)	0 (0)	7 (100)	5 (71.4)	2 (28.6)	0 (0)	3 (42.9)*↓	0 (0)	4 (57.1)	0 (0)	5 (71.4)	2 (28.6)	1 (14.3)	1 (14.3)	5 (71.4)
*E. casseliflavus* (*n* = 5)	0 (0)	0 (0)	5 (100)	0 (0)	0 (0)	5 (100)	0 (0)	0 (0)	5 (100)	5 (100)	0 (0)	0 (0)	3 (60)	0 (0)	2 (40)	0 (0)	5 (100)	0 (0)	1 (20)	0 (0)	4 (80)
*E. cecorum* (*n* = 3)	0 (0)	0 (0)	3 (100)	0 (0)	0 (0)	3 (100)	0 (0)	0 (0)	3 (100)	2 (66.7)	1 (33.3)	0 (0)	3 (100)	0 (0)	0 (0)	0 (0)	2 (66.7)	1 (33.3)	0 (0)*↓	0 (0)	3 (100)
*E. durans* (*n* = 3)	0 (0)	0 (0)	3 (100)	0 (0)	0 (0)	3 (100)	0 (0)	0 (0)	3 (100)	2 (66.7)	1 (33.3)	0 (0)	2 (66.7)	0 (0)	1 (33.3)	1 (33.3)	1 (33.3)	1 (33.3)	3 (100)*↑	0 (0)	0 (0)
Total (*n* = 145)	0 (0)	1 (0.7)	144 (99.3)	0 (0)	0 (0)	145 (100)	5 (3.4)	0 (0)	140 (96.6)	116 (80)	29 (20)	0 (0)	106 (73.1)	0 (0)	39 (26.9)	26 (17.9)	90 (62.1)	29 (20)	43 (29.7)	10 (6.9)	92 (63.5)
*Enterococcus* spp. (*n*)	Chloramphenicol					Erythromycin				High level gentamicin			Vancomycin			Teicoplanin			Linezolid		
	R	I		S		R	I		S	R	I	S	R	I	S	R	I	S	R	I	S
*E. columbae* (*n* = 50)	11 (22)* ↑	8 (16)		31 (62)		33 (66)	2 (4)		15z (30)	1 (2)	0 (0)	49 (98)	5 (10)	6 (12)	39 (78)	39 (78)	0 (0)	11 (22)	0 (0)	1 (2)	49 (98)
*E. hirae* (*n* = 30)	1 (3.3)	3 (10)		26 (86.7)		6 (20)*↓	4 (13.3)		20 (66.7)	1 (3.3)	0 (0)	29 (96.7)	11 (36.7)	4 (13.3)	15 (50)	20 (66.7)	0 (0)	10 (33.3)	0 (0)	0 (0)	30 (100)
*E. faecium* (*n* = 17)	0 (0)	2 (11.8)		15 (88.2)		6 (35.3)	7 (41.2)		4 (23.5)	2 (11.8)	0 (0)	15 (88.2)	3 (17.6)	4 (23.5)	10 (58.8)	7 (41.2)*↓	0 (0)	10 (58.8)	0 (0)	3 (17.6)	14 (82.4)
*E. faecalis* (*n* = 17)	2 (11.8)	8 (47.1)		7 (41.2)		14 (82.4)*↑	2 (11.8)		1 (5.9)	0 (0)	0 (0)	17 (100)	2 (11.8)	14 (82.4)	1 (5.9)	17 (100)	0 (0)	0 (0)	0 (0)	0 (0)	17 (100)
*E. gallinarum* (*n* = 13)	0 (0)	0 (0)		13 (100)		10 (76.9)	1 (7.7)		2 (15.4)	0 (0)	0 (0)	13 (100)	4 (30.8)	8 (61.5)	1 (7.7)	12 (92.3)	0 (0)	1 (7.7)	0 (0)	0 (0)	13 (100)
*E. mundti* (*n* = 7)	0 (0)	0 (0)		7 (100)		1 (14.3)*↓	0 (0)		6 (85.7)	0 (0)	0 (0)	7 (100)	0 (0)	1 (14.3)	6 (85.7)	3 (42.9)*↓	0 (0)	4 (57.1)	0 (0)	0 (0)	7 (100)
*E. casseliflavus* (*n* = 5)	0 (0)	0 (0)		5 (100)		0 (0)*↓	4 (80)		1 (20)	0 (0)	0 (0)	5 (100)	2 (40)	2 (40)	1 (20)	3 (60)	0 (0)	2 (40)	0 (0)	0 (0)	5 (100)
*E. cecorum* (*n* = 3)	1 (33.3)* ↑	0 (0)		2 (66.7)		1 (33.3)	1 (33.3)		1 (33.3)	0 (0)	0 (0)	3 (100)	0 (0)	1 (33.3)	2 (66.7)	3 (100)	0 (0)	0 (0)	0 (0)	1 (33.3)	2 (66.7)
*E. durans* (*n* = 3)	0 (0)	0 (0)		3 (100)		1 (33.3)	0 (0)		2 (66.7)	0 (0)	0 (0)	3 (100)	1 (33.3)	0 (0)	2 (66.7)	2 (66.7)	0 (0)	1 (33.3)	0 (0)	0 (0)	3 (100)
Total (*n* = 145)	15 (10.3)	21 (14.5)		109 (75.2)		72 (49.7)	21 (14.5)		52 (35.9)	4 (2.8)	0 (0)	141 (97.2)	28 (19.3)	40 (27.6)	77 (53.1)	106 (73.1)	0 (0)	39 (26.9)	0 (0)	5 (3.4)	140 (96.6)

* significantly higher or lower percentage of resistant strains at α = 0.05. AUG: amoxicillin/clavulanic acid (20/10 μg); AP: ampicillin (10 μg); PG: penicillin (10 μg); ENF: enrofloxacin (5 μg); DXT: doxycycline (30 μg): TEC: tetracycline (30 μg); NI: nitrofurantoin (300 μg); C: chloramphenicol (30 μg); E: erythromycin (15 μg); GM: high level gentamicin (120 μg); VA: vancomycin (30 μg); T: teicoplanin (30 μg); LZD: linezolid (30 μg)

A total of 42.1% (61/145) isolates showed resistance or intermediate susceptibility to glycopeptides. Most of them belonged to *E. faecalis* (26.3%), *E. hirae* (21.3%), *E. gallinarum* (19.7%), *E. columbae* (16.4%), *E. faecium* (6.6%), *E. casseliflavus* (4.9%), and *E. cecorum, E. durans, E. mundtii* (1.6%). The resistance to enrofloxacin/doxycycline/erythromycin/ teicoplanin predominated among pigeon *Enterococcus*. No resistance to 13 antibiotics was found among 6.9% isolates (Table 7), however there were no pigeon lofts with isolates susceptible to all tested antibiotics.

**Table 7:** Patterns of resistance to antibiotics observed in commensal enterococci (*n* = 145) isolated from racing pigeons

Number of antibiotics	Antibiotic combinations (number of resistant isolates)	Total % (n) of resistant isolates
9	Penicillin/Enrofloxacin/Doxycycline/Nitrofurantoin/Chloramphenicol/Tetracycline/Erythromycin/Teicoplanin/Vancomycin (1)	0.7% (1)
8	Enrofloxacin/Doxycycline/Nitrofurantoin/Tetracycline/Erythromycin/Teicoplanin/High level gentamicin/Vancomycin (1)	2.1% (3)
	Enrofloxacin/Doxycycline/Nitrofurantoin/Chloramphenicol/Tetracycline/Erythromycin/Teicoplanin/Vancomycin (1)	
	Penicillin/Enrofloxacin/Doxycycline/Nitrofurantoin/Tetracycline/Erythromycin/Teicoplanin/Vancomycin (1)	
7	Enrofloxacin/Doxycycline/Nitrofurantoin/Tetracycline/Erythromycin/Teicoplanin/Vancomycin (3)	4.1% (6)
	Enrofloxacin/Doxycycline/Nitrofurantoin/Tetracycline/Teicoplanin/High level gentamicin/Vancomycin (1)	
	Enrofloxacin/Doxycycline/Tetracycline/Erythromycin/Teicoplanin/High level gentamicin/Vancomycin (1)	
	Penicillin/Enrofloxacin/Doxycycline/Tetracycline/Erythromycin/Teicoplanin/Vancomycin (1)	
6	Enrofloxacin/Doxycycline/Nitrofurantoin/Tetracycline/Teicoplanin/Vancomycin (2)	5.5% (8)
	Enrofloxacin/Doxycycline/Nitrofurantoin/ChloramphenicoL/Erythromycin/Teicoplanin (1)	
	Penicillin/Enrofloxacin/Doxycycline/Nitrofurantoin/Erythromycin/Teicoplanin (1)	
	Enrofloxacin/Doxycycline/Nitrofurantoin/Erythromycin/Teicoplanin/Vancomycin (1)	
	Enrofloxacin/Doxycycline/Tetracycline/Erythromycin/Teicoplanin/Vancomycin (1)	
	Enrofloxacin/Doxycycline/Chloramphenicol/Tetracycline/Teicoplanin/Vancomycin (1)	
	Doxycycline/Nitrofurantoin/Tetracycline/Teicoplanin/High level gentamicin/Vancomycin (1)	
5	Enrofloxacin/Doxycycline/Chloramphenicol/Erythromycin/Teicoplanin (8)	17.2% (25)
	Enrofloxacin/Doxycycline/Nitrofurantoin/Erythromycin/Teicoplanin (5)	
	Enrofloxacin/Doxycycline/Tetracycline/Teicoplanin/Vancomycin (4)	
	Enrofloxacin/Doxycycline/Tetracycline/Erythromycin/Teicoplanin (3)	
	Enrofloxacin/Doxycycline/Erythromycin/Teicoplanin/Vancomycin (3)	
	Enrofloxacin/Doxycycline/Nitrofurantoin/Tetracycline/Teicoplanin (1)	
	Enrofloxacin/Doxycycline/Chloramphenicol/Tetracycline/Teicoplanin (1)	
4	Enrofloxacin/Doxycycline/Erythromycin/Teicoplanin (31)	29% (42)
	Enrofloxacin/Doxycycline/Nitrofurantoin/Teicoplanin (6)	
	Enrofloxacin/Doxycycline/Teicoplanin/Vancomycin (2)	
	Enrofloxacin/Doxycycline/Chloramphenicol/Teicoplanin (1)	
	Enrofloxacin/Nitrofurantoin/Tetracycline/Vancomycin (1)	
	Doxycycline/Tetracycline/Teicoplanin/Vancomycin (1)	
3	Enrofloxacin/Doxycycline/Teicoplanin (11)	16.6% (24)
	Doxycycline/Erythromycin/Teicoplanin (6)	
	Enrofloxacin/Nitrofurantoin/Erythromycin (3)	
	Enrofloxacin/Nitrofurantoin/Tetracycline (1)	
	Doxycycline/Chloramphenicol/Teicoplanin (1)	
	Doxycycline/Teicoplanin/Vancomycin (1)	
	Penicillin/Doxycycline/Teicoplanin (1)	
2	Enrofloxacin/Nitrofurantoin (6)	5.5% (8)
	Doxycycline/Teicoplanin (2)	
1	Enrofloxacin (12)	12.4% (18)
	Nitrofurantoin (6)	
0	0	6.9% (10)

### Prevalence of vancomycin resistance genes

Of all *Enterococcus* spp., 25.5% (37/145) isolates were positive for at least one *van* gene. Among *van*-positive enterococci, 13.5% (5/37) of isolates contained more than one *van* gene: 1 isolate contained three *van* genes (*E. columbae*: *van*B, *van*C1, *van*C2-C3), 4 isolates contained two *van* genes (3 *E. hirae*: *van*C1, *van*C2-C3, and 1 *E. columbae*: *van*B, *van*C1). *Van*C1 gene was carried significantly more often by *E. gallinarum* (*p* < 0.001), *vanC2-C3* gene by *E. casseliflavus* (*p* < 0.001) and *E. hirae* (*p* = 0.011). There were no significant differences (*p* = 0.277) in the occurrence of *van*B between enterococcal species (Table 8). Four pigeon lofts (30.8%), were free from *van*-positive enterococci.

**Table 8:** The prevalence of *Enterococcus*-associated vancomycin resistance genes in healthy racing pigeons

*Enterococcus* spp.	No. of isolates	Number (%) of isolates harbouring the *van* gene			
		*van*A	*van*B	*van*C1	*van*C2-C3
*E. columbae*	50	0	5 (10%)	8 (16%)	1 (2%)
*E. hirae*	30	0	0	4 (13.3%)	6 (20%)*↑
*E. faecium*	17	0	0	2 (11.8%)	0 (0%)
*E. faecalis*	17	0	0	2 (11.8%)	0 (0%)
*E. gallinarum*	13	0	0	9 (69.2%)*↑	0 (0%)
*E. mundtii*	7	0	0	0 (0%)	0 (0%)
*E. casseliflavus*	5	0	0	0 (0%)	5 (100%)*↑
*E. cecorum*	3	0	0	2 (66.7%)	0 (0%)
*E. durans*	3	0	0	0 (0%)	0 (0%)
Total	145	0	5 (3.4%)	27 (18.6%)	12 (8.3%)

* significantly higher percentage of isolates with *van* gene at α = 0.05

Among van-positive enterococci, 59.5% (22/37) of them were resistant or intermediate susceptible to vancomycin and belonged to *E. gallinarum* (36.4%; 8/22), *E. casseliflavus* (18.2%; 4/22), *E. hirae* (13.6%; 3/22), *E. columbae* (13.6%; 3/22), *E. faecalis* (9.2%; 2/22), *E. faecium, E. cecorum* (4.5%; 1/22). Among *van*-positive isolates, 54.1% (20/37) of them were resistant or intermediate susceptible to glycopeptide antibiotics (vancomycin, teicoplanin) and belonged to *E. gallinarum* (45%; 9/20), *E. casseliflavus* (15%; 3/20), *E. hirae*,* E. faecalis, E. columbae* (10%; 2/20), *E. faecium, E. cecorum* (5%; 1/20).

## Discussion

Despite the literature describing gut microbiota in pigeons, there is a little information regarding the enterococcal microbiota and distribution of *Enterococcus* species in domestic pigeons [1, 18]. Most of available works have concerned feral pigeons [2–4, 19, 20]. Evaluation of the cloacal content of racing pigeons in this study revealed a high prevalence of *Enterococcus* isolation. Our results contradicted the previous findings suggesting that *Enterococcus* species were naturally associated with humans and generally were rarely found in pigeons [2]. A total of 65–67% enterococcal species were correctly identified using API & Multiplex PCR as well as API & 16S sequencing. Jackson at al [18]*.* obtained higher agreement for poultry or swine isolates by API and PCR. In our study, Multiplex PCR and sequencing were the most adequate methods used for identification of pigeon *Enterococcus* species and the accuracy of these methods was approx. 97%. The highest accuracy (compared to the gold standard) was observed for Multiplex PCR, a bit lower for 16S sequencing and much lower for API.

According to the literature, the 16S rRNA gene sequence-based results used for bacteria identification should be analysed by two or more databases due to the difficulties in interpretation [21]. According to the recommendations the obtained results were compared with two freely available, quality-controlled, web-based public databases: GenBank and EzTaxon. Although both databases attributed equally all *Enterococcus* isolates to the same species, there was moderate to poor agreement between databases in the classification of isolates to a given species and did not seem to differ between enterococcal species. Our results support the findings, that the analysis by GenBank combined with EzTaxon is more discriminative than analysis by GenBank alone [21].

Similarly to other studies [1, 18] we showed that *E. columbae* predominated in intestinal microbiota of racing pigeons. On the other hand, there were pigeon lofts lacking *E. columbae* species. *Enterococcus columbae* was not found in city pigeons in Egypt [4]. Interestingly, among domestic birds only pigeons have their own specific *Enterococcus* species. *Enterococcus columbae* has not been recognized as a component of normal gut microbiota in other species. This phenomenon has not been clarified yet, however the certain features of the pigeon microbiota may have an impact because of their rudimentarily developed caeca. The main findings support the fact that pigeon enterococcal gut microbiota is composed of host-specific bacteria [1, 22]. The frequent occurrence of *E. hirae* in this study contrasts with the findings of other authors [1, 4] who did not find *E. hirae* in pigeon intestines. Besides of pigeons, *E. hirae* is one of the most commonly encountered enterococcal species in poultry [23–25]. According to the literature, *E. faecalis* followed by *E. faecium* are the most prevalent enterococcal species colonizing the gastrointestinal tract in humans, poultry and wild birds [8, 12, 23]. We found that *E. faecalis* and *E. faecium* were the third most common *Enterococcus* species identified in racing pigeons*.* In contrast to domestic pigeons, it seems that *E. faecalis* and *E. faecium* largely dominated the enterococcal intestinal microbiota in feral pigeons [3, 19]. Screening of the intestinal enterococcal microbiota revealed that *E. gallinarum*, originally described in chickens, was rarely found in racing pigeons [23, 24]. *Enterococcus cecorum* was less commonly found in pigeon lofts, and occurred irregularly but usually after race season. Interestingly, among the known *Enterococcus* species, only *E. cecorum* was described as the cause of enterococcal infections in racing pigeons [6, 7]. Recently, *E. cecorum* commonly considered as a part of physiologic intestinal microbiota of animals, has grown into one of the leading bacterial cause of lameness in broiler chickens [7–9, 24]. Similarly to Zigo et al. [22] *E. mundtii* was a rarely identified in racing pigeons. However, other authors observed *E. mundtii* more than twice as prevalent in feral pigeons [3]. This study revealed lower prevalence of *E. durans* racing pigeons than in poultry and feral pigeons [1, 4, 22–25]. It seems that *Enterococcus raffinosus* is absent in racing pigeons, but can occur in feral pigeons [4]. In contrast to poultry, *Enterococcus avium* and *Enterococcus aquimarinus* were not found in pigeons [2, 3, 22–24]. Based on the obtained results and literature data we deduced that pigeon microbiota is composed by similar enterococcal species (except *E. columbae*) that have been demonstrated in poultry [23, 25].

In our study the enterococcal intestinal microbiota in racing pigeons was the most abundant with *Enterococcus* species (up to 7) after the race season and *E. columbae*, *E. faecium*, *E. hirae* were the most prevalent. Before racing season the microbiota was composed by 4 *Enterococcus* species, with *E. columbae* and *E. hirae* as dominant. Similarly to our study Zigo et al. [22] identified more *Enterococcus* species in intestinal microbiota after race season. In contrast to our results, they recorded 3 dominant *Enterococcus* species before as well as after race season in pigeons. In our study, 2–5 *Enterococcus* species were found during the race season and *E. columbae*, *E. faecium*, *E. faecalis*, *E. gallinarum* predominated. Our results were consistent with the mentioned authors who showed that intestinal microbiota during the race season was dominated by *E. faecalis, E. faecium, E. columbae* [22]. We concluded that the race season may affect the presence of commensal bacteria in pigeons and may have impact on the differences in enterococcal gut microbiome composition between pigeon lofts. Moreover, some geographical differences in the occurrence of enterococci were observed.

To our best knowledge, this is the first study which provides differences in biochemical characteristics between intestinal *Enterococcus* species originated from racing pigeons. So far most studies concerning pigeons enterococci have provided biochemical properties only for *E. columbae*. Similarly to the literature [1, 5], few *E. columbae* isolates revealed activity of arginine dihydrolase (ADH), pyroglutamic acid arylamidase (PYRA), and ability to metabolize glycogen (GLYG). In contrast to Baele at al. [1], many *E. columbae* isolates were positive for alkaline phosphatase (PAL), and trehalose (TRE). We found that *Enterococcus columbae* was very similar to *E. cecorum* in terms of biochemical properties with exception of 7/32 characteristics (βNAG, HIP, PUL, LARA, MβDG, TAG, βMAN).

So far, among studies concerning pigeons enterococcal virulence factors were examined only in wood pigeons (*Columba palumbus*) [19]. In this study we confirmed the presence of 6 different virulence genes in *Enterococcus* isolated from intestinal microbiota of racing pigeons. A higher prevalence of *gelE, asa1* and *ace* was in line with other studies concerning enterococci in farm animals [9, 16] but not with the results for enterococci isolated from food-stuffs [26]. The lower detection rate of *cylA* and *esp* in racing pigeons was in near agreement with poultry isolates [9, 16, 27]. Incidences of *esp* detection in enterococci isolated from cattle and pigs were higher than in our study [16, 27]. We confirmed that pigeon enterococci did not contain hyaluronidase gene. The absence of *hyl* was noted also in enterococci obtained from wild game meat and farm animals [16, 17, 27]. In contrast to pigeon, *hyl* was often reported in human, wild bird, food and water isolates [13, 15, 24, 28]. Jung et al. [9] found *hyl* among single pathogenic *E. cecorum* from poultry.

The study revealed that small number of racing pigeon enterococci produced gelatinase, but 66.7% *gelE* genes were silent. Among virulence genes found in *E. columbae*, *gelE* was the predominant one. High occurrence of *efaA*, *ace* and gelatinase activity was observed in *E. faecalis*. Our findings differed from those of Martín et al. [19] who did not confirm silent *gelE* genes in enterococci from wood pigeons. Moreover, they did not find any of the examined virulence genes in *E. columbae*. Our results corroborate similar findings by authors who described the highest gelatinase activity in *E. faecalis* [14, 19]. Among enterococci from feral pigeons, mainly *E. faecium* hydrolyzed gelatinase [4]*.*

Enterococci harbouring virulence factors are more likely to cause infections than those without them. However, *E. cecorum* and *E. columbae* retrieved from infection case in racing pigeon were negative for virulence factors [7]. None of virulence factors was found significantly more often in the pathogenic poultry isolates compared to the commensal ones [9]. More data are needed on the distribution of enterococcal virulence factors for the purpose of establishing their role in the pathogenesis of *Enterococcus*-associated diseases in pigeons.

The prevalence of antimicrobial resistant enterococci in racing pigeons investigated in this study was very high. A total of 93.1% isolates showed resistance to at least one antimicrobial, and 29% were resistant to 4 antibiotics. On the contrary, Radimersky et al. [3] reported a lower number of isolates resistant to at least one antibiotic in feral pigeons. The most frequent resistance to tetracycline was observed in strains obtained from feral or racing pigeons and from wild birds [3, 12, 18, 28]. We concluded that frequent resistance to enrofloxacin and doxycycline may be associated with a common usage of these antibiotics in domestic pigeons. According to the actual legislation*,* only several veterinary medicinal products used in pigeons have been authorised for marketing within the territory of Poland, including amoxicillin, enrofloxacin, norfloxacin, doxycycline*.* On the other hand resistance to antibiotics which are not approved for use in pigeons (e.g. aminoglycosides, macrolides, phenicols) or even in animals (glycopeptide antibiotics) may suggest acquisition of resistance from other sources. The resistance to vancomycin was more frequent than observed in enterococci from feral pigeons in Brazil, Czech Republic [2, 3], and 5-times less frequent than in feral pigeons in Egypt [4]. Our study revealed significant differences between some enterococcal species in susceptibility to different antibiotics *(*doxycycline, nitrofurantoin, chloramphenicol, erythromycin, teicoplanin). Similarly to other authors [18] we showed more frequent resistance of *E. columbae* to enrofloxacin. We concluded that the most VRE belonged to *E. hirae* and *E. columbae*, while the most HLGR isolates belonged to *E. faecium*. On the contrary, Butaye et al. [18] did not find VRE in pigeons, whereas HLGR were represented mainly by *E. faecalis*. For the first time enterococci from racing pigeon were screened against linezolid. In contrast to feral pigeons [4], no LRE were found in racing pigeons.

This study, to the best of our knowledge provides, for the first time, detailed vancomycin resistance genotypes of a variety of enterococci isolated from racing pigeons. In contrast to the meat of wild game animals (including pigeons), wild birds, feral pigeons [3, 4, 17, 28], racing pigeons did not harbour *van*A-enterococci and only sporadically *van*B. Similarly to our results, other authors did not find *van*A-mediated glycopeptide resistance in enterococci isolated from fecal samples in pigeons [29]. However *van*B predominated in feral pigeons [4]. Out of the vancomycin resistance genes tested, *van*C1 and *van*C2-C3 predominated in racing pigeons*.* Vancomycin resistance caused by the *van*C genes predominated in *Enterococcus* isolates in chickens [4]. Our data confirm significantly higher prevalence of *van*C2-C3 among *E. casseliflavus* and *E. hirae*. However in chickens the *van*C gene predominated among *E. faecium* [4]. We concluded that vancomycin resistance of *E. columbae* was caused by the *van*C1, *van*B or *van*C1-C2 genes.

## Conclusions

This study revealed that 9 different *Enterococcus* species: *E. columbae, E. hirae, E. faecalis, E. faecium, E. gallinarum, E. mundtii, E. casseliflavus, E. cecorum, E. durans* inhabit theintestinal microbiota in racing pigeons. Our results indicated that the composition of the enterococcal intestinal microbiota of racing pigeons differ between pigeon lofts and may vary depending on the race season. *Enterococcus* species showed differences in relation to 68.8% (22/32) biochemical characteristics. The host-specific *E. columbae* harboured *gelE*, *ace*, *asa1, van*C1, *van*B, *van*C2-C3, and showed significant resistance to chloramphenicol. Racing pigeons could serve as source of important *Enterococcus* species as well as the potential spreaders of multidrug-resistant strains, virulence genes through their close contact with humans, backyard animals, wild birds and their ability to fly over many kilometers during competition flights. To the best of our knowledge, this is the first study on the distribution of *Enterococcus* species in racing pigeons, their biochemical properties, the occurrence of enterococcal virulence factors, and vancomycin resistance genes.

## Methods

### Bacterial strains

Cloacal swabs were collected from 179 clinically healthy mature racing pigeons residing in 13 different lofts (pigeon houses) belonged to Polish Association of Racing Pigeon Breeders (PZHGP) and located in central Poland: Mazovia voivodeship (11/13 lofts; 52° 13′ 0″ N, 21° 0′ 0″ E), in southern Poland: Silesia (1/13; 50°20′N 19°00′E), Świętokrzyskie voivodeship (1/13; 50°45′N 20°46′E). Cloacal samples were collected by inserting the sterile viscose-tipped sticks (Deltalab, Spain) into the cloaca and swabbing the mucosal wall by gentle rotation. The sticks were moistened with sterile water (Aqua pro Injectione; Polpharma, Poland) just before sampling to prevent the dry swab from causing mucosal damage. After sampling, each stick was placed into sterile tube and immediately transferred to the laboratory. At first cloacal swabs were inoculated onto the selective medium (Enterococcosel agar; Graso Poland). The full 1 μl loop of colonies that grow on Enterococcosel agar and resembled enterococcal colony morphology (small, beige with strong black halos) were passaged onto the Columbia agar enriched with 5% sheep blood (Graso Poland). The plates were incubated at 37 °C for 24 h in CO_2_ enriched atmosphere (5% CO_2_). Single colonies with typical enterococcal morphology and characteristics (type of hemolysis, Gram-positive, catalase negative) were further identified by using API test and molecular methods.

### Identification of *Enterococcus* species by biochemical test

The biochemical identification was performed by using API rapid ID 32 STREP (bioMérieux, France) in accordance with the manufacturer’s instructions.

### Identification of *Enterococcus* species by multiplex PCR

Multiplex PCR reactions were performed with the primers complementary to the internal fragment of *sodA* gene encoding manganese-dependent superoxide dismutase [30]*.* Additional file 7 shows the primer sequences, annealing temperatures, products size, positive controls. Bacterial DNA was extracted using boiling method. PCRs were performed in 25 μl volume containing 12.5 μl DreamTaq PCR Master Mix (Thermo Fisher Scientific Inc., USA), 0.3 μl of each primer (50 pmol/μl), 3 μl DNA and PCR-clean water. The thermocycler program consisted of initial denaturation 95 °C for 5 min, followed by 30 cycles of 95 °C for 30 s, 55 °C for 1 min (or 60 °C), 72 °C for 1 min, and a final extension step at 72 °C for 7 min*.*

### ***Identification*** of Enterococcus species ***by sequencing*** of partial ***16S*** ribosomal RNA

The partial section of the 16S rRNA gene was amplified using PCR with the following primer pair 91E: 5′-TCAAAGGAATTGACGGGG-3′ and 13B: 5′-CCGGGAACGTATTCACCG-3′ which produced a 492-bp fragment [31]. The forward primer was used in the sequencing reactions. Thermocycling conditions were as follows: initial denaturation at 95 °C for 3 min, followed by 38 cycles: denaturation at 93 °C for 30 s, annealing at 49 °C for 30s, extension at 72 °C for 1 min, followed by final extension step 72 °C for 7 min. The obtained sequences were compared with two public databases: 1) GenBank which was searched using NCBI BLAST https://blast.ncbi.nlm.nih.gov/Blast.cgi, and 2) EzTaxon database https://www.ezbiocloud.net/ [32]. Sequencing results were verified by single PCR using *sodA* species-specific primers (gold standard) which confirmed the final results of identification [30]. In cases of discrepancy between 16S rRNA-sequencing and species-specific PCR, the partial *sodA* sequences were then used for sequence analysis. Two phylogenetic trees were constructed using the Neighbor-Joining method (MEGA X software) according to 16S rRNA gene sequence and *sodA* gene sequence evolutionary distances among *Enterococcus* strains [33].

### Virulence factors

*Enterococcus* isolates were tested for the presence of 7 genes encoding virulence factors: *asa1* (aggregation substance), *gelE* (gelatinase), *hyl* (hyaluronidase), *esp* (enterococcal surface protein), *cylA* (cytolysin), *efaA* (endocarditis antigen), *ace* (collagen-binding protein), using three duplex PCRs (*asa1/gelE, cylA/esp, efaA/ace*) and one single PCR (*hyl*) [34]. *Enterococcus faecalis* ATCC 2921 was used as positive control for *asa1 gelE; Enterococcus faecalis* ATCC 51299 was used as positive control *esp, cylA, efaA, ace.* Archival DNA sample extracted from *Enterococcus* strain served as a positive control for *hyl*.

The production of gelatinase by *Enterococcus* was tested in phenotypic assay. The 24 h bacterial cultures were stab-inoculated down the center of Difco Nutrient Gelatin tubes (BD, USA). The inoculated tubes with isolates, as well as with positive (*Staphylococcus aureus* ATCC 25923) and negative (*Escherichia coli* 25922) controls, and non-inoculated tube were incubated at 37 °C. Results were recorded at 1, 7, 10, and 14 day of incubation.

### Antibiotic susceptibility

All isolates were screened for susceptibility to a panel of 13 antibiotics by using disc diffusion method. The following antibiotic-containing discs (Graso, Poland) were applied: amoxicillin/clavulanic acid (AUG 20/10 μg), ampicillin (AP 10 μg), penicillin (PG 10 μg), enrofloxacin (ENF 5 μg), doxycycline (DXT 30 μg), tetracycline (TEC 30 μg), nitrofurantoin (NI 300 μg), chloramphenicol (C 30 μg), erythromycin (E 15 μg), high-level gentamicin (GM 120 μg), vancomycin (VA 30 μg), teicoplanin (T 30 μg), linezolid (LZD 30 μg). *Staphylococcus aureus* ATCC 25923, *Enterococcus faecalis* ATCC 51299, ATCC 29212 served as control strains. The inhibition zones were interpreted as sensitive (S), intermediate (I), resistant (R) according to the CLSI guidelines [35, 36].

### Vancomycin resistance genes: *van*A, *van*B, *van*C1, *van*C2-C3

Two single PCRs for *van*A, *van*B and one duplex PCR for *van*C1, *van*C2-C3 were applied [37]. Thermocycling conditions were: initial denaturation step of 94 °C for 3 min, 35 cycles of denaturation at 94 °C for 1 min, annealing at 56 °C for 1 min, extension at 72 °C for 1 min, followed by an elongation step at 72 °C for 10 min [16]. Reference strains from ATCC collection served as positive controls: *E. faecium* 700,221 (*van*A), *E. faecalis* 51,299 (*van*B), *E. gallinarum* 700,425 (*van*C1), *E. casseliflavus* 700,327 (*van*C2–3).

### Statistical analysis

Numerical variables were presented as a median, inter quartile range (IQR) and range. Categorical variables were given as counts and percentages, which were then compared between groups using a Pearson chi-square test. As more than 2 groups were compared a chi-square test was an omnibus test. Therefore, when it yielded significant result, a post hoc analysis was performed according to Markowski and Markowski [38]. Ninety five percent confidence intervals (CI 95%) for proportions were calculated using the Wilson’s score method. Prevalence was compared between *Enterococcus* species using z-test with the Bonferroni correction for multiple comparisons (i.e. *p*-value of a single test × 3 comparison performed). Agreement between two bases (GenBank and EzTaxon) was assessed using the Gwet’s AC1. The cut-off for classification of a strain into a particular species was set at 98.65% [39]. The Gwet’s AC1 statistics was chosen to control for kappa paradoxes resulting from the unbalanced marginal distribution of results of the two gene bases [40]. Agreement between categorical results with more than two categories was determined with Cohen’s kappa statistics. Both AC1 and Cohen’s kappa were interpreted as: < 0 poor agreement, 0–0.2 slight agreement, 0.21–0.40 fair agreement, 0.41–0.60 moderate agreement, 0.61–0.80 substantial agreement, 0.81–1.0 almost perfect agreement. All statistical tests were two-sided. A significance level (α) was set at 0.05. Statistical analysis was performed in Statistica 13 (TIBCO Software) except for Cohen’s kappa which was computed in IBM SPSS Statistics 24 and Gwet’s AC1 which was calculated in Microsoft Excel.

## Supplementary information

**Additional file 1. **Prevalence of *Enterococcus* spp. within 13 pigeon lofts.

**Additional file 2. **Phylogenetic tree based on 16S gene sequence analysis, showing the relationships among 145 commensal *Enterococcus* species from racing pigeons. Five isolates no. 83, 86, 99, 129, 133 were finally identified (*sodA* PCR and sequencing) as *E. mundtii*, *E. durans*, *E. faecium,* and *E. hirae* (2x) respectively, although 16S-sequencing recognized them as *E. columbae*. The percentage of replicate trees in which the associated taxa clustered together in the bootstrap test (1000 replicates) are shown next to the branches. The tree is drawn to scale, with branch lengths in the same units as those of the evolutionary distances used to infer the phylogenetic tree. The evolutionary distances were computed using the Maximum Composite Likelihood method and are in the units of the number of base substitutions per site.

**Additional file 3. **Phylogenetic tree built by the Neighbor-Joining method using *sodA* gene sequences and showing phylogenetic relationships of commensal *Enterococcus* isolates (*n* = 145) from racing pigens. The percentage of replicate trees in which the associated taxa clustered together in the bootstrap test (1000 replicates) are shown next to the branches. The tree is drawn to scale, with branch lengths in the same units as those of the evolutionary distances used to infer the phylogenetic tree. The evolutionary distances were computed using the Maximum Composite Likelihood method and are in the units of the number of base substitutions per site.

**Additional file 4. **Comparison of biochemical test (API rapid ID 32 STREP) and Multiplex PCR in identification of *Enterococcus* species in racing pigeons.

**Additional file 5. **Comparison of sequencing and biochemical test (API rapid ID 32 STREP) in identification of *Enterococcus* species in racing pigeons.

**Additional file 6. **The sensitivity, specificity of three tests (API, Multiplex PCR and sequencing) in identification of *Enterococcus* species in pigeons.

**Additional file 7.** Sequences of PCR primers, products size, and positive controls used in this study. Reference: Jackson et al. 2004 [19].

## Data Availability

Supporting data are included within the article and its Additional files 1-7. The datasets used and analyzed during the current study are available from the corresponding author on reasonable request.

## Abbreviations

HLGR: High-level of gentamicin resistance
LRE: Linezolid-resistant enterococci
VRE: Vancomycin-resistant enterococci

## References

[1] Baele M., Devriese L.A., Butaye P., Haesebrouck F. (2002). Composition of enterococcal and streptococcal flora from pigeon intestines. Journal of Applied Microbiology.

[2] da Silva V^|^acirc;nia L^|^uacute;cia, Ca^|^ccedil;ador Nat^|^aacute;lia C^|^acirc;ndido, da Silva Carolina dos Santos Fernandes, Fontes Cl^|^aacute;udia Oliveira, Garcia Gizele Duarte, Nicoli Jacques Robert, Diniz Cl^|^aacute;udio Galuppo (2012). Occurrence of Multidrug-Resistant and Toxic-Metal Tolerant Enterococci in Fresh Feces from Urban Pigeons in Brazil. Microbes and Environments.

[3] Radimersky T, Frolkova P, Janoszowska D, Dolejska M, Svec P, Roubalova E, Cikova P, Cizek A, Literak I (2010). Antibiotic resistance in faecal bacteria (*Escherichia coli*, *Enterococcus* spp.) in feral pigeons. J Appl Microbiol.

[4] Osman KM, Badr J, Orabi A, Elbehiry A, Saad A, Ibrahim MDS, Hanafy MH (2019). Poultry as a vector for emerging multidrug resistant Enterococcus spp.: first report of vancomycin (van) and the chloramphenicol-florfenicol (cat-fex-cfr) resistance genes from pigeon and duck faeces. Microb Pathog.

[5] Devriese LA, Ceyssens K, Rodrigues UM, Collins MD (1990). *Enterococcus columbae*, a species from pigeon intestines. FEMS Microbiol Lett.

[6] Brash M, Slavic D (2011). An unusual case of *Enterococcus cecorum* septicemia in a racing pigeon. Anim Health Lab Newsl Univ Guelph.

[7] Jung A, Teske L, Rautenschlein S (2014). *Enterococcus cecorum* infection in a racing pigeon. Avian Dis.

[8] Stępień-Pyśniak D, Marek A, Banach T, Adaszek Ł, Pyzik E, Wilczyński J, Winiarczyk S (2016). Prevalence and antibiotic resistance of *Enterococcus* strains isolated from poultry. Acta Vet Hung.

[9] Jung A, Metzner M, Ryll M (2017). Comparison of pathogenic and non-pathogenic *Enterococcus cecorum* strains from different animal species. BMC Microbiol.

[10] Stenzel T, Bancerz-Kisiel A, Tykałowski B, Smiałek M, Pestka D, Koncicki A (2014). Antimicrobial resistance in bacteria isolated from pigeons in Poland. Pol J Vet Sci.

[11] Poeta P, Costa D, Sáenz Y, Klibi N, Ruiz-Larrea F, Rodrigues J, Torres C (2005). Characterization of antibiotic resistance genes and virulence factors in faecal enterococci of wild animals in Portugal. J Vet Med B Infect Dis Vet Public Health.

[12] Stępień-Pyśniak D, Hauschild T, Nowaczek A, Marek A, Dec M (2018). Wild birds as a potential source of known and novel multilocus sequence types of antibiotic-resistant *Enterococcus faecalis*. J Wildl Dis.

[13] Vankerckhoven V, Van Autgaerden T, Vael C, Lammens C, Chapelle S, Rossi R, Jabes D, Goossens H (2004). Development of a multiplex PCR for the detection of *asa1, gelE, cylA, esp*, and *hyl* genes in enterococci and survey for virulence determinants among European hospital isolates of *Enterococcus faecium*. J Clin Microbiol.

[14] Poeta P, Costa D, Klibi N, Rodrigues J, Torres C (2006). Phenotypic and genotypic study of gelatinase and beta-haemolysis activities in faecal enterococci of poultry in Portugal. J Vet Med B Infect Dis Vet Public Health.

[15] Sidhu JP, Skelly E, Hodgers L, Ahmed W, Li Y, Toze S (2014). Prevalence of enterococcus species and their virulence genes in fresh water prior to and after storm events. Environ Sci Technol.

[16] Iweriebor BC, Obi LC, Okoh AI (2015). Virulence and antimicrobial resistance factors of *Enterococcus* spp. isolated from fecal samples from piggery farms in Eastern Cape, South Africa. BMC Microbiol.

[17] Guerrero-Ramos E, Cordero J, Molina-González D, Poeta P, Igrejas G, Alonso-Calleja C, Capita R (2016). Antimicrobial resistance and virulence genes in enterococci from wild game meat in Spain. Food Microbiol.

[18] Butaye P, Baele M, Devriese LA, Haesebrouck F (2002). Comparison of susceptibility to antimicrobials of the enterococcal species isolated from pigeons (*Columba livia*). Microb Drug Resist.

[19] Martín M, Gutiérrez J, Criado R, Herranz C, Cintas LM, Hernández PE (2006). Genes encoding bacteriocins and their expression and potential virulence factors of enterococci isolated from wood pigeons (*Columba palumbus*). J Food Prot.

[20] Blanco-Peña K, Esperón F, Torres-Mejía AM, de la Torre A, de la Cruz E, Jiménez-Soto M (2017). Antimicrobial resistance genes in pigeons from public parks in Costa Rica. Zoonoses Public Health.

[21] Park KS, Ki CS, Kang CI, Kim YJ, Chung DR, Peck KR, Song JH, Lee NY (2012). Evaluation of the GenBank, EzTaxon, and BIBI services for molecular identification of clinical blood culture isolates that were unidentifiable or misidentified by conventional methods. J Clin Microbiol.

[22] Zigo F, Takáč L, Zigová M, Takáčová J (2017). Changes in Bacterial Microflora in Young Carrier Pigeons during the Race Season. Int J Avian Wildlife Biol.

[23] Devriese L. A., Van De Kerckhove A., Kilpper-Balz R., Schleifer K. H. (1987). Characterization and Identification of Enterococcus Species Isolated from the Intestines of Animals. International Journal of Systematic Bacteriology.

[24] Dolka Beata, Gołębiewska–Kosakowska Mariola, Krajewski Krzysztof, Kwieciński Piotr, Nowak Tomasz, Szubstarski Jarosław, Wilczyński Jarosław, Szeleszczuk Piotr (2017). Occurrence of Enterococcus spp. in poultry in Poland based on 2014–2015 data. Medycyna Weterynaryjna.

[25] Devriese LA, Hommez J, Wijfels R, Haesebrouck F (1991). Composition of the enterococcal and streptococcal intestinal flora of poultry. J Appl Bacteriol.

[26] Trivedi K, Cupakova S, Karpiskova R (2011). Virulence factors and antibiotic resistance in enterococci isolated from food-stuffs. Vet Med.

[27] Seputiene V, Bogdaite A, Ruzauskas M, Suziedeliene E (2012). Antibiotic resistance genes and virulence factors in *Enterococcus faecium* and *Enterococcus faecalis* from diseased farm animals: pigs, cattle and poultry. Pol J Vet Sci.

[28] Yahia HB, Chairat S, Hamdi N, Gharsa H, Ben Sallem R, Ceballos S, Torres C, Ben SK (2018). Antimicrobial resistance and genetic lineages of faecal enterococci of wild birds: emergence of vanA and vanB2 harbouring *Enterococcus faecalis*. Int J Antimicrob Agents.

[29] Devriese LA, Ieven M, Goossens H, Vandamme P, Pot B, Hommez J, Haesebrouck F (1996). Presence of vancomycin-resistant enterococci in farm and pet animals. Antimicrob Agents Chemother.

[30] Jackson CR, Fedoka-Cray PJ, Barett JB (2004). Use of a genus- and species-specific multiplex PCR for identication of enterococci. J Clin Microbiol.

[31] Relman DA, Persing DH, Smith TF, Tenover FC, White TJ (1993). Universal bacterial 16S rDNA amplification and sequencing. Diagnostic molecular microbiology – principles and applications.

[32] Yoon SH, Ha SM, Kwon S, Lim J, Kim Y, Seo H, Chun J (2017). Introducing EzBioCloud: a taxonomically united database of 16S rRNA and whole genome assemblies. Int J Syst Evol Microbiol.

[33] Kumar S, Stecher G, Li M, Knyaz C, Tamura K (2018). MEGA X: MEGA X: Molecular evolutionary genetics analysis across computing platforms. Mol Biol Evol.

[34] Martín-Platero AM, Valdivia E, Maqueda M, Martínez-Bueno M (2009). Characterization and safety evaluation of enterococci isolated from Spanish goats' milk cheeses. Int J Food Microbiol.

[35] CLSI (2018). Performance standards for antimicrobial disk and dilution susceptibility tests for Bacteria isolated from animals. 4th ed. CLSI supplement VET08.

[36] CLSI (2019). Performance standards for antimicrobial susceptibility testing. 29th ed. CLSI supplement M100.

[37] Nam S, Kim MJ, Park C, Park JG, Maeng PJ, Lee GC (2013). Detection and genotyping of vancomycin-resistant *Enterococcus* spp. by multiplex polymerase chain reaction in Korean aquatic environmental samples. Int J Hyg Environ Health.

[38] Markowski EP, Markowski CA (2009). A systematic method for teaching post hoc. Analysis of Chi-Square tests. Decis Sci J Innov Educ.

[39] Kim M, Oh HS, Park SC, Chun J (2014). Towards a taxonomic coherence between average nucleotide identity and 16S rRNA gene sequence similarity for species demarcation of prokaryotes. Int J Syst Evol Microbiol.

[40] Wongpakaran N, Wongpakaran T, Wedding D, Gwet KL (2013). A comparison of Cohen's kappa and Gwet's AC1 when calculating inter-rater reliability coefficients: a study conducted with personality disorder samples. BMC Med Res Methodol.

