# Supplementary material for: Prevalence, antibiotic susceptibility and virulence factors of Enterococcus species in racing pigeons (Columba livia f. domestica)
Source: BMC Vet Res. 2020 Jan 8;16:7. doi: 10.1186/s12917-019-2200-6 (PMC6947970; doi:10.1186/s12917-019-2200-6)
Supplement: Supplementary file 1 — Additional file 1. Prevalence of Enterococcus spp. within 13 pigeon lofts. [file 12917_2019_2200_MOESM1_ESM.doc]

**Additional file 1 Prevalence of *Enterococcus* spp. within 13 pigeon lofts.**

| Loft no. | Sampling season | Voivodeship | No. of enterococcal isolates | No. of  enterococcal  species | Risk of *Enterococcus* isolation | *Enterococcus* species found within each pigeon loft |
| --- | --- | --- | --- | --- | --- | --- |
| 6 | September | Masovia | 15 | 7 | 100% | *E. hirae* (33.3%), *E. faecalis* (20%), *E. mundtii* (20%), *E. faecium* (6.7%),  *E. gallinarum* (6.7%), *E. casseliflavus* (6.7%), *E. durans* (6.7%) |
| 9 | September | Masovia | 24 | 6 | 73.3% | *E. columbae* (45.8%), *E. hirae* (25%), *E. faecium* (12.5%), *E. casseliflavus* (8.3%), *E. mundtii* (4.2%), *E. durans* (4.2%) |
| 2 | November | Masovia | 17 | 5 | 59.1% | *E. columbae* (41.2%), *E. hirae* (29.4%), *E. gallinarum* (17.6%), *E. mundtii* (5.9%), *E. casseliflavus* (5.9%) |
| 5 | May | Silesia | 9 | 5 | 100% | *E. faecium* (44.4%), *E. columbae* (22.2%), *E. faecalis* (11.1%), *E. gallinarum* (11.1%), *E. casseliflavus* (11.1%) |
| 4 | June | Masovia | 20 | 4 | 90.9% | *E. columbae* (45%), *E. faecalis* (35%), *E. faecium* (10%), *E. gallinarum* (10%) |
| 11 | January | Masovia | 13 | 4 | 92.9% | *E. hirae* (38.5%), *E. faecium* (23.1%), *E. faecalis* (23.1%), *E. columbae* (15.4%) |
| 7 | February | Masovia | 11 | 4 | 91.7% | *E. hirae* (54.5%), *E. columbae* (18.2%), *E. mundtii* (18.2%), *E. durans* (9.1%) |
| 10 | February | Masovia | 9 | 4 | 100% | *E. columbae* (55.6%), *E. faecalis* (22.2%), *E. gallinarum* (11.1%), *E. cecorum* (11.1%) |
| 3 | January | Masovia | 7 | 4 | 36.8% | *E. hirae* (42.9%), *E. columbae* (28.6%), *E. faecium* (14.3%), *E. gallinarum* (14.3%) |
| 1 | October | Masovia | 12 | 3 | 60% | *E. columbae* (75%), *E. cecorum* (16.7%), *E. gallinarum* (8.3%) |
| 12 | June | Masovia | 5 | 2 | 100% | *E. gallinarum* (60%), *E. faecium* (40%) |
| 8 | May | Masovia | 2 | 2 | 100% | *E. columbae* (50%), *E. faecalis* (50%) |
| 13 | November | Świętokrzyskie | 1 | 1 | 100% | *E. faecium* (100%) |
