# Supplementary figures and images for: Prevalence, antibiotic susceptibility and virulence factors of Enterococcus species in racing pigeons (Columba livia f. domestica)
Source: BMC Vet Res. 2020 Jan 8;16:7. doi: 10.1186/s12917-019-2200-6 (PMC6947970; doi:10.1186/s12917-019-2200-6)

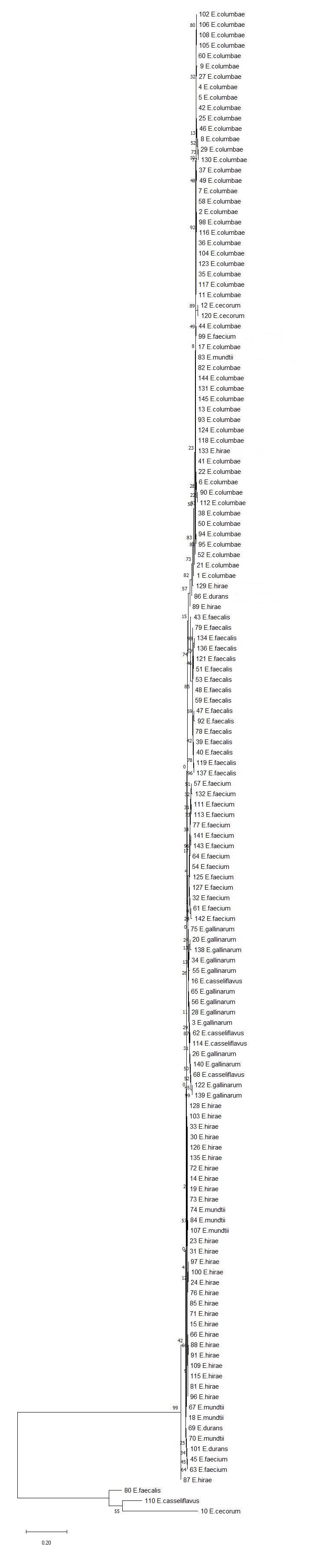

Supplement: Supplementary file 2 — Additional file 2. Phylogenetic tree based on 16S gene sequence analysis, showing the relationships among 145 commensal Enterococcus species from racing pigeons. Five isolates no. 83, 86, 99, 129, 133 were finally identified (sodA PCR and sequencing) as E. mundtii, E. durans, E. faecium, and E. hirae (2x) respectively, although 16S-sequencing recognized them as E. columbae. The percentage of replicate trees in which the associated taxa clustered together in the bootstrap test (1000 replicates) are shown next to the branches. The tree is drawn to scale, with branch lengths in the same units as those of the evolutionary distances used to infer the phylogenetic tree. The evolutionary distances were computed using the Maximum Composite Likelihood method and are in the units of the number of base substitutions per site. [file 12917_2019_2200_MOESM2_ESM.jpg]

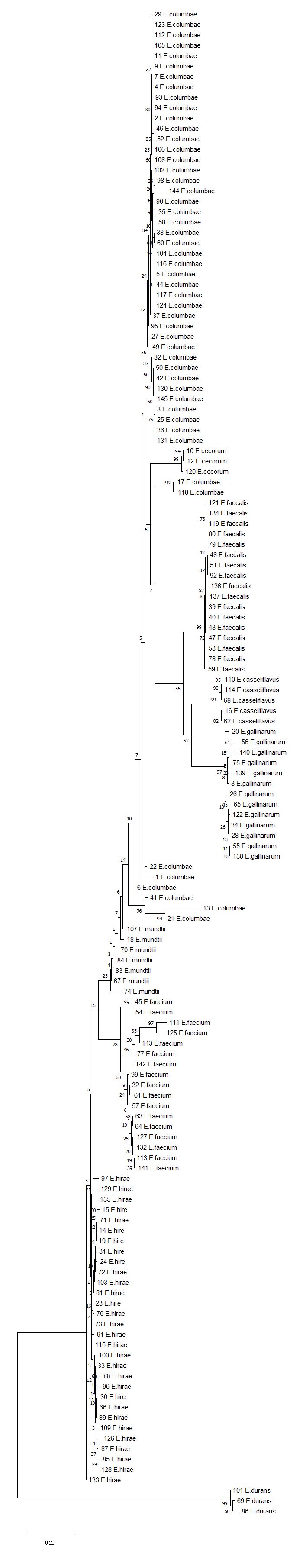

Supplement: Supplementary file 3 — Additional file 3. Phylogenetic tree built by the Neighbor-Joining method using sodA gene sequences and showing phylogenetic relationships of commensal Enterococcus isolates (n = 145) from racing pigens. The percentage of replicate trees in which the associated taxa clustered together in the bootstrap test (1000 replicates) are shown next to the branches. The tree is drawn to scale, with branch lengths in the same units as those of the evolutionary distances used to infer the phylogenetic tree. The evolutionary distances were computed using the Maximum Composite Likelihood method and are in the units of the number of base substitutions per site. [file 12917_2019_2200_MOESM3_ESM.jpg]
