# Supplementary material for: Prevalence, antibiotic susceptibility and virulence factors of Enterococcus species in racing pigeons (Columba livia f. domestica)
Source: BMC Vet Res. 2020 Jan 8;16:7. doi: 10.1186/s12917-019-2200-6 (PMC6947970; doi:10.1186/s12917-019-2200-6)
Supplement: Supplementary file 4 — Additional file 4. Comparison of biochemical test (API rapid ID 32 STREP) and Multiplex PCR in identification of Enterococcus species in racing pigeons. [file 12917_2019_2200_MOESM4_ESM.doc]

**Additional file 4 Comparison of biochemical test (API rapid ID 32 STREP) and Multiplex (*sodA*) PCR in identification of *Enterococcus* species in racing pigeons.**

|  |  | Multiplex PCR | | | | | | | Total  (n) |
| --- | --- | --- | --- | --- | --- | --- | --- | --- | --- |
|  | *Enterococcus* spp. | *E.  hirae* | *E. faecium* | *E.  faecalis* | *E. gallinarum* | *E.  casseliflavus* | *E.  cecorum* | *E.  durans* |  |
| API | *E. hirae* | 20 | 0 | 0 | 0 | 0 | 0 | 2 | 22 |
|  | *E. faecium* | 0 | 7 | 0 | 1 | 1 | 0 | 0 | 9 |
|  | *E. faecalis* | 1 | 1 | 13 | 0 | 0 | 0 | 0 | 15 |
|  | *E. gallinarum* | 0 | 7 | 3 | 12 | 0 | 1 | 0 | 23 |
|  | *E. casseliflavus* | 6 | 2 | 1 | 0 | 4 | 1 | 1 | 15 |
|  | *E. cecorum* | 0 | 0 | 0 | 0 | 0 | 1 | 0 | 1 |
|  | *E. durans* | 3 | 0 | 0 | 0 | 0 | 0 | 0 | 3 |
| Total (n) | | 30 | 17 | 17 | 13 | 5 | 3 | 3 | 88 |

Total n=88; without *E. columbae* (n=50) and *E. mundtii* (n=7). Cohen’s kappa = 0.566 (CI 95%: 0.450, 0.682) – moderate agreement.

Green indicates agreement in species identification between PCR and API; yellow indicates a lack of agreement.
