# Supplementary material for: Prevalence, antibiotic susceptibility and virulence factors of Enterococcus species in racing pigeons (Columba livia f. domestica)
Source: BMC Vet Res. 2020 Jan 8;16:7. doi: 10.1186/s12917-019-2200-6 (PMC6947970; doi:10.1186/s12917-019-2200-6)
Supplement: Supplementary file 5 — Additional file 5. Comparison of sequencing and biochemical test (API rapid ID 32 STREP) in identification of Enterococcus species in racing pigeons. [file 12917_2019_2200_MOESM5_ESM.doc]

**Additional file 5 Comparison of sequencing and biochemical test (API rapid ID 32 STREP) in identification of *Enterococcus* species in racing pigeons.**

|  |  | 16S Sequencing | | | | | | | Total (n) |
| --- | --- | --- | --- | --- | --- | --- | --- | --- | --- |
|  | *Enterococcus* spp. | *E.  hirae* | *E.  faecium* | *E.  faecalis* | *E.  gallinarum* | *E.  casseliflavus* | *E.  cecorum* | *E.  durans* |  |
| API | *E. hirae* | 19 | 0 | 0 | 0 | 0 | 0 | 1 | 20 |
|  | *E. faecium* | 0 | 7 | 0 | 1 | 1 | 0 | 0 | 9 |
|  | *E. faecalis* | 1 | 1 | 13 | 0 | 0 | 0 | 0 | 15 |
|  | *E. gallinarum* | 0 | 7 | 3 | 12 | 0 | 1 | 0 | 23 |
|  | *E. casseliflavus* | 5 | 1 | 1 | 0 | 4 | 1 | 1 | 13 |
|  | *E. cecorum* | 0 | 0 | 0 | 0 | 0 | 1 | 0 | 1 |
|  | *E. durans* | 3 | 0 | 0 | 0 | 0 | 0 | 0 | 3 |
| Total (n) | | 28 | 16 | 17 | 13 | 5 | 3 | 2 | 84 |

Total n=84; without *E. columbae* (n=50) and *E. mundtii* (n=7) and isolates (n=4) that were identified in the sequencing as *E. columbae,* but in API as different species. Cohen’s kappa = 0.589 (CI 95%: 0.471, 0.707) – moderate agreement

Green indicates agreement in species identification between sequencing and API; yellow indicates a lack of agreement.
