# Supplementary material for: Prevalence, antibiotic susceptibility and virulence factors of Enterococcus species in racing pigeons (Columba livia f. domestica)
Source: BMC Vet Res. 2020 Jan 8;16:7. doi: 10.1186/s12917-019-2200-6 (PMC6947970; doi:10.1186/s12917-019-2200-6)
Supplement: Supplementary file 6 — Additional file 6. The sensitivity, specificity of three tests (API, Multiplex PCR and sequencing) in identification of Enterococcus species in pigeons. [file 12917_2019_2200_MOESM6_ESM.doc]

**Additional file 6 The sensitivity*,* specificity of three tests (API, Multiplex PCR and sequencing) in identification of *Enterococcus* species in pigeons.**

| *Enterococcus* species | n | API rapid ID 32 STREP  (**bioMérieux, France)** | | Multiplex *sodA*-PCR | | Sequencing (16S rRNA) | |
| --- | --- | --- | --- | --- | --- | --- | --- |
|  |  | Se (95% CI) | Sp (95% CI) | Se (95% CI) | Sp (95% CI) | Se (95% CI) | Sp (95% CI) |
| *E. columbae* | 50 | 0%  (0%, 7.1%) | 100%  (96.1%, 100%) | 100%  (92.9%, 100%) | 100%  (96.1%, 100%) | 100%  (92.9%, 100%) | 94.7%  (88.3%, 97.7%) |
| *E. hirae* | 30 | 66.7%  (48.8%, 80.8%) | 98.3%  (93.9%, 99.5%) | 100%  (88.6%, 100%) | 100%  (96.8%, 100%) | 93.3%  (78.7%, 98.2%) | 100%  (96.8%, 100%) |
| *E. faecium* | 17 | 41.2%  (21.6%, 64.0%) | 97.7%  (93.3%, 99.2%) | 100%  (81.6%, 100%) | 100%  (97.1%, 100%) | 94.1%  (73.0%, 99.0%) | 100%  (97.1%, 100%) |
| *E. faecalis* | 17 | 76.5%  (52.7%, 90.4%) | 98.4%  (94.5%, 99.6%) | 100%  (81.6%, 100%) | 100%  (97.1%, 100%) | 100%  (81.6%, 100%) | 100%  (97.1%, 100%) |
| *E. gallinarum* | 13 | 92.3%  (66.7%, 98.6%) | 90.2%  (83.9%, 94.2%) | 100%  (77.2%, 100%) | 100%  (97.2%, 100%) | 100%  (77.2%, 100%) | 100%  (97.2%, 100%) |
| *E. mundtii* | 7 | 0%  (0%, 35.4%) | 100%  (97.3%, 100%) | 100%  (64.6%, 100%) | 100%  (97.2%, 100%) | 85.7%  (48.7%, 97.4%) | 100%  (97.3%, 100%) |
| *E. casseliflavus* | 5 | 80.0%  (37.6%, 96.4%) | 71.4%  (63.5%, 78.3%) | 100%  (56.6%, 100%) | 100%  (97.3%, 100%) | 100%  (56.6%, 100%) | 100%  (97.3%, 100%) |
| *E. cecorum* | 3 | 33.3%  (6.1%, 79.2%) | 92.3%  (86.7%, 95.6%) | 100%  (43.9%, 100%) | 100%  (97.4%, 100%) | 100%  (43.9%, 100%) | 100%  (97.4%, 100%) |
| *E. durans* | 3 | 0%  (0%, 56.1%) | 96.5%  (92.0%, 98.5%) | 100%  (43.9%, 100%) | 100%  (97.4%, 100%) | 66.7%  (20.8%, 93.9%) | 100%  (97.4%, 100%) |

Se –Sensitivity; Sp-Specificity
