# Supplementary material for: Prevalence, antibiotic susceptibility and virulence factors of Enterococcus species in racing pigeons (Columba livia f. domestica)
Source: BMC Vet Res. 2020 Jan 8;16:7. doi: 10.1186/s12917-019-2200-6 (PMC6947970; doi:10.1186/s12917-019-2200-6)
Supplement: Supplementary file 7 — Additional file 7. Sequences of PCR primers, products size, and positive controls used in this study. Reference: Jackson et al. 2004 [19]. [file 12917_2019_2200_MOESM7_ESM.docx]

**Additional file 7**  Sequences of PCR primers, products size, and positive controls used in this study. Reference: Jackson et al. 2004 [30].

| Strains (positive control) | Primers | Sequences (5’–3’) | Annealingtemp. (°C) | Product  size (bp) |
| --- | --- | --- | --- | --- |
| *E. avium* ATCC 14025 | AV1 | GCTGCGATTGAAAAATATCCG | 55 | 368 |
|  | AV2 | AAGCCAATGATCGGTGTTTTT |  |  |
| *E. casseliflavus* ATCC 700327 | CA1 | TCCTGAATTAGGTGAAAAAAC | 55 | 288 |
|  | CA2 | GCTAGTTTACCGTCTTTAACG |  |  |
| *E. cecorum* ATCC 43198 | CE1 | AAACATCATAAAACCTATTTA | 55 | 371 |
|  | CE2 | AATGGTGAATCTTGGTTCGCA |  |  |
| *E. columbae* (our laboratory strain) | CO1 | GAATTTGGTACCAAGACAGTT | 55 | 284 |
|  | CO2 | GCTAATTTACCGTTATCGACT |  |  |
| *E. durans* ATCC 6056 | DU1 | CCTACTGATATTAAGACAGCG | 55 | 295 |
|  | DU2 | TAATCCTAAGATAGGTGTTTG |  |  |
| *E. faecalis* ATCC 29212 | FL1 | ACTTATGTGACTAACTTAACC | 55 | 360 |
|  | FL2 | TAATGGTGAATCTTGGTTTGG |  |  |
| *E. faecium* ATCC 700221 | FM1 | GAAAAAACAATAGAAGAATTAT | 55 | 215 |
|  | FM2 | TGCTTTTTTGAATTCTTCTTTA |  |  |
| *E. gallinarum* ATCC 700425 | GA1 | TTACTTGCTGATTTTGATTCG | 55 | 173 |
|  | GA2 | TGAATTCTTCTTTGAAATCAG |  |  |
| *E. hirae* ATCC 10541 | HI1 | CTTTCTGATATGGATGCTGTC | 55 | 187 |
|  | HI2 | TAAATTCTTCCTTAAATGTTG |  |  |
| *E. mundtii* (our laboratory strain) | MU1 | CAGACATGGATGCTATTCCATCT | 60 | 98 |
|  | MU2 | GCCATGATTTTCCAGAAGAAT |  |  |
| *E. raffinosus* ATCC 49464 | RF1 | GTCACGAACTTGAATGAAGTT | 55 | 287 |
|  | RF2 | AATGGGCTATCTTGATTCGCG |  |  |
